# Green Synthesis of Stable Spherical Monodisperse Silver Nanoparticles Using a Cell-Free Extract of *Trichoderma reesei*

**DOI:** 10.3390/ma15020481

**Published:** 2022-01-09

**Authors:** Orlin Gemishev, Marinela Panayotova, Gospodinka Gicheva, Neli Mintcheva

**Affiliations:** 1 Faculty of Biology, Sofia University “St. Kl. Ohridski”, 1000 Sofia, Bulgaria; o.gemishev@uni-sofia.bg; 2 Department of Chemistry, University of Mining and Geology, 1700 Sofia, Bulgaria; marichim@mgu.bg (M.P.); g.gicheva@mgu.bg (G.G.)

**Keywords:** silver nanoparticles, green method, cell-free water extract, *Trichoderma reesei*, antibacterial properties

## Abstract

In the current study, a green method for the preparation of silver nanoparticles (AgNPs) is presented as an alternative to conventional chemical and physical approaches. A biomass of *Trichoderma reesei* (*T. reesei*) fungus was used as a green and renewable source of reductase enzymes and metabolites, which are capable of transforming Ag^+^ ions into AgNPs with a small size (mainly 2–6 nm) and narrow size distribution (2–25 nm). Moreover, extracellular biosynthesis was carried out with a cell-free water extract (CFE) of *T. reesei*, which allows for facile monitoring of the bioreduction process using UV–Vis spectroscopy and investigation of the effect of experimental conditions on the transformation of Ag^+^ ions into AgNPs, as well as the simple isolation of as-prepared AgNPs for the study of their size, morphology and antibacterial properties. In continuation to our previous results about the influence of media on *T. reesei* cultivation, the amount of biomass used for CFE preparation and the concentration of Ag^+^ ion solution, herein, we present the impact of temperature (4, 20, 30 and 40 °C), agitation and time duration on the biosynthesis of AgNPs and their properties. A high stability of AgNPs in aqueous colloids was observed and attributed to the capping effect of the biomolecules as shown by the zeta potential (−49.0/−51.4 mV) and confirmed by the hydrodynamic size of 190.8/116.8 nm of AgNPs.

## 1. Introduction

Silver nanoparticles (AgNPs) are widely applied in different areas, such as food industry, water disinfection, textile industry, environment protection and catalysis, and their scope of use is gradually increasing [1,2,3,4]. AgNPs are desired components in formulations for biomedicine due to their antibacterial [5,6], antifungal [7], antiviral [8], anti-inflammatory [9], anti-angiogenic [10] and anticancer activities [11]. Additionally, AgNPs are favorable candidates for target drug delivery and wound healing in pharmacology [4,12,13]. Such a wide application of AgNPs requires tuning of their physical characteristics, such as size, size distribution, shape and morphology, which can be achieved by using different methods of preparation. AgNPs can be obtained using various physical methods, such as ball milling [14]; aerosol techniques [15]; microwave, UV and IR irradiation [16,17,18]; arc discharge [19]; metal vaporization [20]; and laser ablation [21]. A variety of chemical methods have been developed for AgNP formation, including chemical reduction [22,23], electrochemical reduction [24], solvent extraction reduction [25], alcohol reduction [26], the micro-emulsion technique [27] and sonochemical processing [28]. In both types of approaches, for the stabilization of AgNPs, capping agents are added, which prevent nanoparticle agglomeration. Generally, chemical reagents and organic solvents accumulate a waste and cause negative environmental impacts.

Recently, the green synthesis of AgNPs attracted the attention of researchers and is now widely studied. AgNP preparation via the use of various plant extracts [29,30,31,32,33], actinomycetes, yeasts, algae, fungi and viruses has been proposed [1,32,34,35]. Among microbial-facilitated AgNP formation, the use of fungi is considered as a prospective approach since fungi grow fast and, thus, ensure a higher productivity of nanoparticles (compared to other microbes), easy handling and processing. These advantages are combined with the desirable characteristics of AgNPs, for example, tunable size and high monodispersity [12,35,36,37]. Fungi are able to biosynthesize AgNPs via the intracellular and/or extracellular reduction of metal ions by cell enzymes, proteins, metabolites, sugars etc. [12,36,37]. Intracellular synthesis requires further operations to harvest the produced NPs by destroying the cells and separating the nanoparticles. As such, in most studies, extracellular synthesis is carried out, which is based on the ability of the fungal metabolites and enzymes to convert the toxic metal ions for microorganisms into non-toxic atoms that further grow into nanoparticles. Early studies described the attempts of AgNP formation by the use of entire fungi suspension [38,39], while, in later studies, cell-free extract was mainly applied [36,37,40]. The cell-free water extract (CFE) was obtained by washing the mycelia multiple times, followed by its extraction with sterile distilled water for 24 h and further separation of the mycelia [36,37,40]. Seventy percent of fungi that have been investigated in AgNP synthesis are pathogenic for plants, humans and animals [41]. From a safety and economic point of view, precautions are needed to deal with their negative side effects on society and the environment, which lead to the corresponding costs of the research.

In contrast, some *Trichoderma* species are nonpathogenic fungi; therefore, they can be used for a safe and environmentally friendly production of AgNPs. The extracellular biosynthesis of AgNPs has been carried out by using *T. asperellum* [42], *T. viride* [43], *T. atroviride* [44], *T. harzianum* [45] and *T. koningii* [46]. *Trichoderma reesei* is a nonpathogenic, ecofriendly fungus with a high growth rate and high-scale production of extracellular enzymes and proteins [47]. It secretes reductase enzymes that are able to reduce toxic silver ions to nontoxic AgNPs [37,41]. The formation of AgNPs, as well as their properties (size and shape), depends on different parameters, such as the concentration of the silver precursor (i.e., AgNO_3_) and its pH value; reaction time; temperature; and mass transfer conditions, i.e., mixing [33,48]. The used fungus [49,50] and the biomass/sterile distilled water ratio used in the CFE preparation [50,51] are additional factors that are worthy of attention when fungi metabolites and enzymes are used in AgNP synthesis. Some studies are available and report temperature effects on fungal-mediated AgNPs synthesis [45,49,50,51,52,53,54]. However, to the best of our knowledge, such studies have not been conducted for AgNPs obtained by means of *T. reesei* CFE reduction of Ag^+^ ions.

Another factor for AgNP growth is the mass transfer process, which is controlled by the stirring rate of the reaction mixture. In different studies on AgNP production, different fixed stirring rates were used starting from 120 [50] to 200 rpm [49]. In most cases, the syntheses were carried out at 150 rpm, for example, in [45,52,53]. However, investigations on the effect of stirring rate on AgNP formation and properties are very limited [48,49]. To the best of our knowledge, a systematic study on the effect of stirring rate on AgNP production using *T. reesei* CFE and the properties of the produced NPs is not available. As such, we were motivated to explore both factors in our research.

Due to the advantageous features of *T. reesei*, we used this fungus for the extracellular synthesis of AgNPs by applying CFE obtained through the water extraction of carefully washed fungus biomass. We studied the effect of the medium on *T. reesei* cultivation [55], as well as the impact of time, the concentration of the AgNO_3_ precursor and the concentration of CFE on the AgNP formation [56].

In this paper, we applied our previous findings concerning optimized silver nitrate concentration, medium for *T. reesei* cultivation and the amount of biomass used for cell-free water extraction, and, further, we investigated the impact of temperature, reaction time and agitation on the shape, size and stability of the AgNP suspension formed by the bioreduction of Ag+ ions to AgNPs. The AgNPs produced were tested for antibacterial activity toward *Escherichia coli*.

## 2. Materials and Methods

### 2.1. Fungus Cultivation and Synthesis of AgNPs

The *Trichoderma reesei* PF strain was obtained from the collection of the Biotechnology Department, Faculty of Biology, Sofia University “St. Kliment Ohridski”.

A seven-day-old culture of *T. reesei* cultivated on potato dextrose agar at 30 ± 1 °C was used in the study. The fungal biomass was obtained after inoculating the strain in 100 mL of growth media, which contained 2% glucose, 0.1% NH_4_Cl, 0.03% CO(NH_2_)_2_, 0.1% (NH_4_)_2_SO_4_, 0.2% KH_2_PO_4_, 0.03% MgSO_4_ 7H_2_O, 0.04% CaCl_2_ 2H_2_O and 0.1% corn steep liquor. All reagents were dissolved in sterile distilled water. The reagents used for media preparation and fungi cultivation were of p.a. grade, produced by Sigma-Aldrich, St. Louis, MO, USA. The incubation of the fungi was carried out in flasks of 500 cm^3^ for 72 h at 30 ± 1 °C, under continual shaking at 220 rpm. Sterile Whatman No. 1 filter paper was used to separate the fungus biomass from the culture broth. The biomass was washed multiple times (over twenty times) with sterile distilled water. Then, the wet biomass was suspended in sterile distilled H_2_O (10% biomass), and it was extracted for 24 h, under constant shaking at 150 rpm. After that, the utilized fungus biomass was separated by filtration, and the obtained CFE (containing a mixture of fungus metabolites) was used in the further experiments. Silver nitrate was dissolved in the produced CFE to obtain Ag^+^ ions with a concentration of 10 mM.

The formation of AgNPs via Ag^+^ ion reduction with enzyme extract from *T. reesei* fungus was studied at various temperatures, 4, 20, 30 and 40 °C, without stirring. The range was chosen by taking into account the temperature ranges for enzyme production by *T. reesei* [57,58] and the experimental conditions and findings of other authors for nanoparticle synthesis via *T. Trichoderma harzianum* [45] and *Trichoderma viride* [52].

In the experiments aimed at studying the effect of stirring on AgNP extracellular biosynthesis, the mixture was stirred with speeds of 0, 50, 150 and 250 rpm in 500 cm^3^ flasks at 30 ± 1 °C.

All experiments were carried out in dark conditions.

Sample notation is as presented in Table 1.

### 2.2. AgNP Characterization

Formation of AgNPs was monitored from 0 h to 144 h by UV–Vis absorption spectroscopy (on BOECO S-220 UV/VIS spectrophotometer, Hamburg, Germany) at a wavelength from 200 to 600 nm. Four milliliter samples of CFE, containing the produced AgNPs, were taken every 24 h, and their absorbance was recorded. On the 72nd and 144th h, the as-prepared AgNPs were isolated by centrifugation (30 min, 13,300 rpm), washed with distilled water several times and re-suspended to record their spectra.

Inductively coupled plasma-atomic emission spectroscopy (ICP-AES) was used to determine the concentration of silver in samples prepared by isolation of AgNPs from the reaction mixture and their dissolution in acidic solution. AgNPs were isolated from equal volumes of reaction mixture, and the prepared acidic solutions were in equal volumes in order to ensure correct comparison for different conditions.

The synthesized and isolated nanoparticles were characterized by transmission electron microscopy (TEM). An electron microscope, JEOL model JEM 2100, 200 kV (JEOL, Tokyo, Japan), was used. A drop of re-suspended AgNPs was cast on carbon-coated copper grid and dried under ambient conditions before measurement. The dynamic light scattering (DLS) method was applied to determine the zeta potential, Z-average particle size (hydrodynamic size) and polydispersity index (PDI) of the AgNPs prepared under static and rotation conditions by using a Malvern Nano-ZS 90 analyzer (Malvern, Worcestershire, UK). For the FTIR analysis, AgNPs were washed with distilled water and ethanol, dried at 40 °C and mixed with KBr to prepare a pellet. The FTIR spectrum was scanned in the range of 4000–400 cm^−1^ by using a Nicolet 6700 FTIR spectrometer (Thermo Fisher Scientific, Waltham, MA, USA).

### 2.3. Antibacterial Activity of AgNPs

The antibacterial activity of the synthesized AgNPs was investigated against *E. coli* strain 3398 (ordered from Bulgarian National Bank for Industrial Microorganisms and Cell Cultures, Sofia, Bulgaria) by using the agar well diffusion method. Experiments were conducted with AgNPs synthesized for 144 h under stirring (150 rpm) and without stirring (samples Ag-144-150 rpm and Ag-144-0 rpm). *E. coli* strain 3398, applied in the experiments, was pre-grown on a Luria (low salt) agar, containing sodium chloride (0.5 g/L), tryptone (10 g/L) yeast extract (5 g/L) and agar (15 g/L), for 16 h at 37 ± 0.1 °C to obtain cultures in a log phase of growth. A 0.1 mL *E. coli* suspension (10^5^ CFU/mL) was used to prepare bacterial lawns on the surface of individual plates containing Luria agar. Wells of 7 mm diameter were made on the agar. Each well was loaded with different amounts (25, 50, 75 and 100 µL) of produced AgNPs, and distilled water was used as a control. Thus, prepared samples were cultivated for 24 h at 37 ± 0.1 °C, and the inhibition zones were measured (mm). Four replicates of each trial were carried out.

## 3. Results and Discussion

### 3.1. Temperature Dependence of AgNP Formation

**Figure 1:**
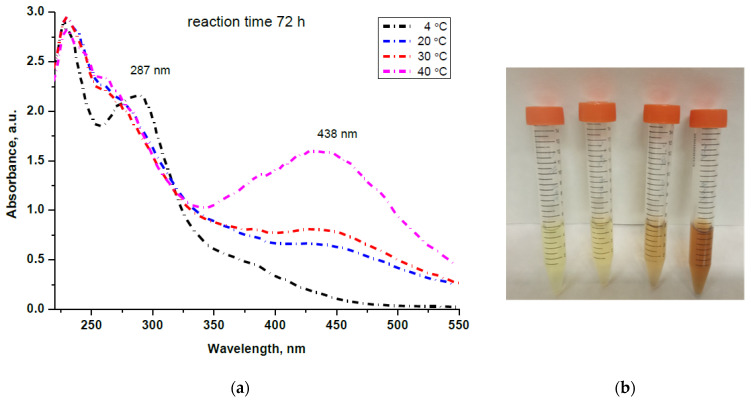
(**a**) UV–Vis spectra taken at 72nd h from the reaction mixtures kept at different temperatures (4, 20, 30 and 40 °C). (**b**) Color change of the reaction mixture for 24 h at 4, 20, 30 and 40 °C from left to right.

The formation of AgNPs by reduction with enzyme extract from *T. reesei* fungi was studied at temperatures of 4, 20, 30 and 40 °C without stirring and monitored using UV–Vis spectroscopy. Based on regular 24 h measurements and our previous experimental results reported elsewhere [56], we chose to examine the reaction mixtures at the 72nd and 144th h of reaction duration and to isolate the AgNPs from the organic liquor for further characterization. Figure 1a depicts the absorption spectra of the reaction mixtures kept at different temperatures for a 72 h reaction time, and Figure 1b visualizes the color changes of the same samples, which were noticeable even on the first day. It can be seen that the intensity of the peak centered at 438 nm, which is assigned to the plasmon resonance of metallic Ag [59], increases with the temperature, and it is well pronounced for the sample obtained at 40 °C.

However, at 4 °C, the rate of the reaction was very slow due to the low enzyme activity at such a temperature [54]. A very slow reduction reaction rate was also observed by Ahluwalia and coauthors [45] at 10 °C when CFE from *Trichoderma harzianum* was used as a reducing agent. A characteristic band at 287 nm for the proteins remained unchanged, and only a shoulder at 360 nm appeared in the spectrum of the sample kept at 4 °C for 72 h (black line). This is the reason why we chose to exclude that sample from the next steps of the analysis.

It is worth mentioning that the dispersion is stable and that further aggregation of AgNPs does not occur until the 144th h of reaction, as a red shift of the absorption band in the UV–Vis spectra was not observed.

**Figure 2:**
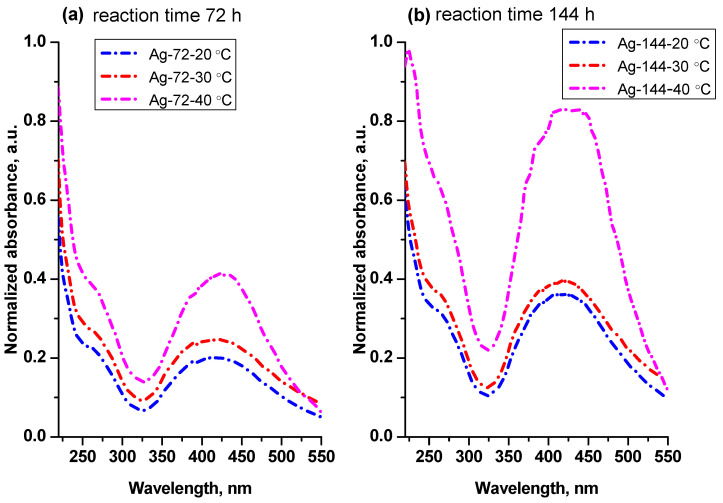
Temperature dependence of AgNP formation at 20, 30 and 40 °C. UV–Vis spectra of aqueous dispersions of AgNPs isolated from the reaction mixture after 72 h reaction time (**a**) and 144 h reaction time (**b**).

The AgNPs formed at the 72 h and 144 h reaction times from the enzyme-induced reaction were isolated, washed with water and re-dispersed in distilled water. Figure 2 shows the absorption spectra of the aqueous dispersions of the AgNPs formed at 20, 30 and 40 °C and isolated from the reaction mixture after 72 h (Figure 2a) and 144 h reaction times (Figure 2b). The absorption bands are observed at 415 nm without a significant shift of the band maximum, suggesting that the particle size and distribution are not drastically changed in the temperature range of 20–40 °C.

Ma and coauthors observed the surface plasmon resonance absorption band at 417 nm for AgNPs produced for 72 h by using CFE from *Penicillium aculeatum* fungus [51]. Rolim et al. observed the surface plasmon resonance absorption band at 410 nm for AgNPs and PEG-capped AgNPs produced by green tea extract biosynthesis [60]. Moreover, some authors found a relationship between the maximum of the absorption band and the NP shape; for example, spherical AgNPs showed a peak around 400 nm, while long-wavelength peaks arose from triangular and hexagonal shapes [48].

Figure 2 also shows that the intensity of the absorption bands increases with the temperature and time, indicating that the amount of AgNPs increases if the reaction is carried out at a higher temperature, as well as for a longer time.

The concentrations of Ag in the aqueous dispersions obtained at different temperatures and reaction times were determined by ICP, and the results are listed in Table 2. ICP data are in good agreement with the UV–Vis results and confirmed that the highest amount of AgNPs was prepared at 40 °C with a 144 h reaction duration.

As it can be seen in Figure 2 and Table 2, the rate of reduction and, correspondingly, the amounts of AgNPs formed at 20 and 30 °C are very close. Ahluwalia and coauthors [45] also observed proximate reaction rates for Ag^+^ ions’ reduction at 20 and 30 °C and a significant increase in the reaction rate when the temperature was increased up to 40 °C. They did not find a notable distinction between the reaction rates at 40 and 50 °C. Ma and coauthors determined 37 °C as the optimal reaction temperature, at which the highest yield of AgNPs was produced by CFE from *Penicillium aculeatum* with a narrow size distribution of NPs [51].

**Figure 3:**
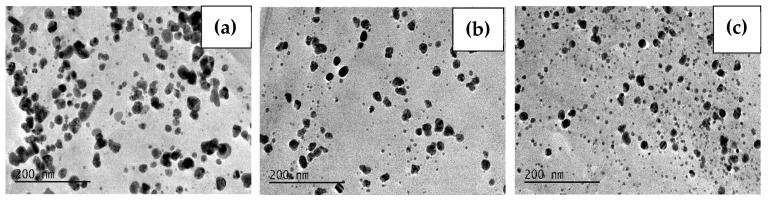
TEM images of the samples (**a**) Ag-72-20 °C, (**b**) Ag-72-30 °C and (**c**) Ag-72-40 °C.

**Figure 4:**
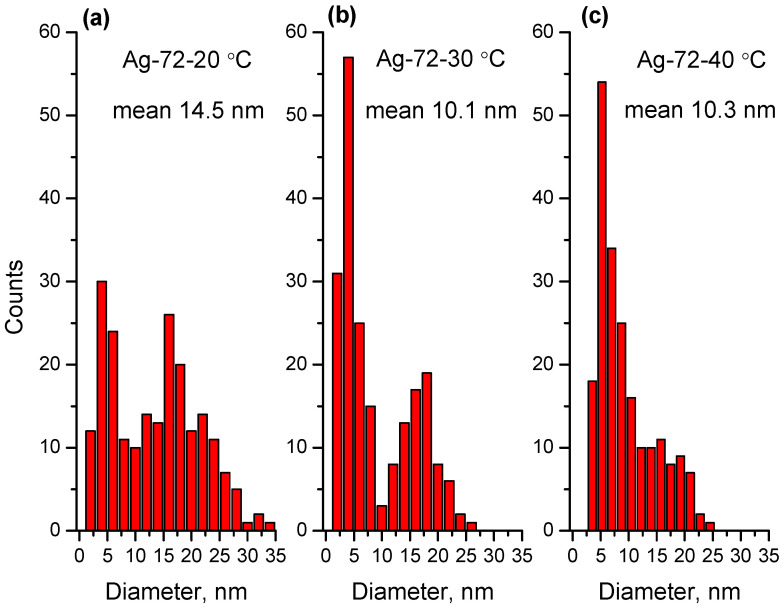
Size distribution for the samples (**a**) Ag-72-20 °C, (**b**) Ag-72-30 °C and (**c**) Ag-72-40 °C.

**Figure 5:**
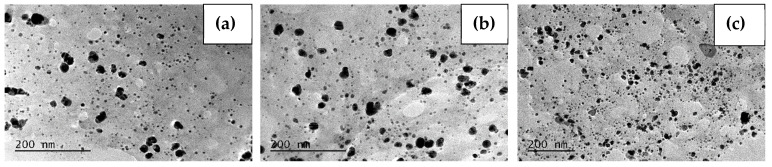
TEM images of the samples (**a**) Ag-144-20 °C, (**b**) Ag-144-30 °C and (**c**) Ag-144-40 °C.

**Figure 6:**
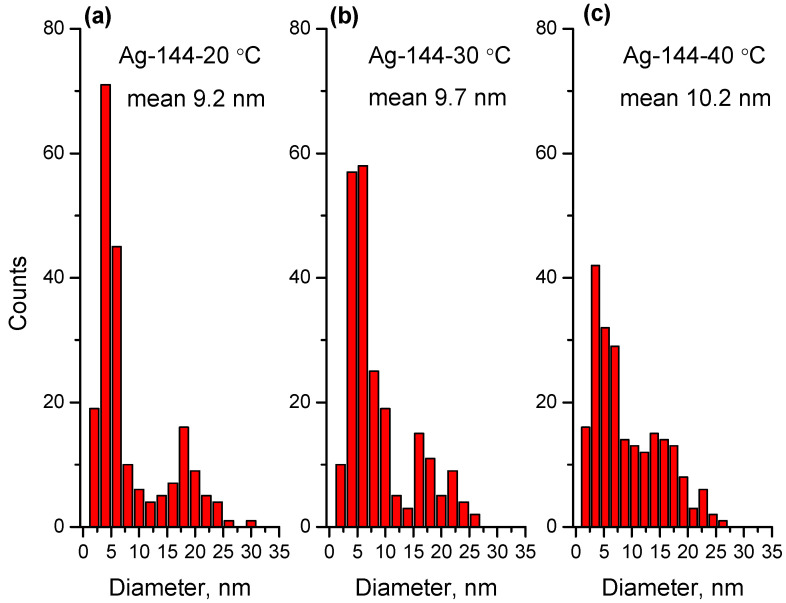
Size distribution for the samples (**a**) Ag-144-20 °C, (**b**) Ag-144-30 °C and (**c**) Ag-144-40 °C.

Furthermore, the morphology and particle size were characterized using TEM. The TEM images and size distribution for the samples Ag-72-20 °C, Ag-72-30 °C and Ag-72-40 °C are presented in Figure 3 and Figure 4, respectively. In Figure 5 and Figure 6, TEM micrographs and size distribution diagrams of the samples Ag-144-20 °C, Ag-144-30 °C and Ag-144-40 °C, respectively, are displayed. The AgNPs show a sphere-like shape and a particle diameter in the range of 2–25 nm (up to 35 nm for Ag-72-20 °C), which is slightly affected by both the temperature and reaction time. In all images (Figure 3 and Figure 5), numerous small nanoparticles (2–6 nm) and bigger ones (15–20 nm) are seen, resulting from a slow formation of particles with different sizes in the presence of CFE. In the case of sample Ag-72-20 °C, nanoparticles with a relatively higher average diameter than those of Ag-72-30 °C and Ag-72-40 °C were observed. A similar effect of temperature on the extracellular synthesis of AgNPs with the water extract of *Rhizopus stolonifer* was observed by Rahim and coauthors. They found large AgNPs (with size 25.89 nm) produced at 20 °C and small monodispersed AgNPs (with size 2.86 nm) synthesized at 40 °C. [54].

At the same time, the average diameters of the AgNPs formed at the three temperatures and isolated at the 144th hour are very similar, and it is confirmed that the temperature (in the range of 20–40 °C) does not play a crucial role in the NP’s size change. Regardless of the temperature, with an increase in the time duration, the AgNPs become unisized. In contrast, Cieśla et al. found that if the temperature increased from 5 to 20 °C, the absorbance maxima shifted toward longer wavelengths corresponding to bigger nanoparticles [48], while Fayaz and coauthors [52] found that the UV–Vis peaks of AgNPs moved to a smaller wavelength with an increase in the temperature (405 nm at 40 °C, 420 nm at 27 °C and 451 nm at 10 °C) when *Trichoderma viride* extract was used. However, we take note that, in their case, the amount of biomass used for the water extraction is 2.5 times higher, which might be crucial for NP growth.

Recently, Liu et al., for the first time, discussed in detail the effect of temperature on particle size based on a microscopic kinetic study. They found that in addition to temperature, the concentration of the Ag^+^ ions is also crucial for nucleation and growth processes during the formation of AgNPs, and they confirmed the conclusion that the size decreases with an increase in the temperature if the Ag^+^ ions are in excess in the system with respect to the reducing agent [61].

In our experiments, the initial concentration of Ag^+^ was kept constant at all temperatures (4, 20, 30 and 40 °C), and it was sufficient for AgNP formation and growth. Previously, we found that less than 10% of Ag^+^ ions were bio-transformed to AgNPs, leaving a sufficient amount of unreduced ions in the liquor, which might be reduced under appropriate conditions. As can be seen in Figure 4, at elevated temperatures (30 and 40 °C), the mean size is smaller than that at 20 °C, which can be explained with the favorable growth of Ag clusters rather than nucleation at lower temperatures. At the same time, at higher temperatures, the reduction rate of Ag^+^ ions is faster, giving more Ag(0) centers, which grow into AgNPs with a smaller size, higher concentration and narrow size distribution [52].

A prolonged keeping of the reaction mixture (144 h) leads to uniform size and size distribution, as shown in Figure 5 and Figure 6. Importantly, after the 72 h, the reaction continued to occur, and more AgNPs were produced, as seen in Figure 2b. As such, we chose a temperature of 40 °C and a duration of 144 h as optimal conditions for the production of AgNPs with a moderate yield and a particle size in the range of 2–25 nm.

### 3.2. Stirring Dependance of AgNP Formation

In order to evaluate the effect of stirring on the rate of AgNP formation, as well as on the size and shape of the AgNPs, synthesis was carried out under static conditions and with continuous stirring with a rotation speed from 50 to 250 rpm. The samples were kept at a temperature of 30 ± 1 °C and processed for UV–Vis measurement, isolation and further characterization at the 72nd and 144th h of reaction duration.

**Figure 7:**
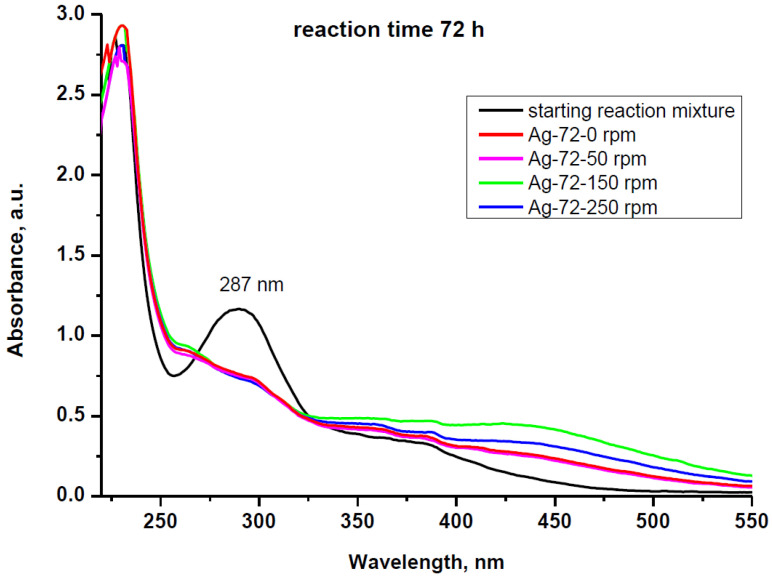
UV–Vis spectra of the starting reaction mixture and the reaction mixtures kept under static conditions (Ag-72-0 rpm) and stirring with different rotation speeds (50, 150 and 250 rpm) for reaction time 72 h. The temperature was kept constant (30 ± 1 °C).

**Figure 8:**
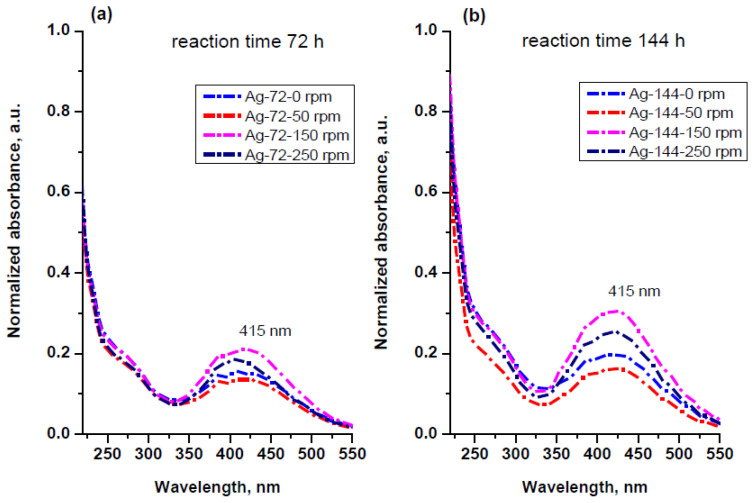
UV–Vis spectra of the aqueous dispersions of AgNPs isolated from the reaction mixtures stirred at 0, 50, 150 and 250 rpm after (**a**) 72 h reaction time and (**b**) 144 h reaction time.

The absorbance spectra of the reaction mixtures taken 72 h after keeping the samples at different stirring conditions are shown in Figure 7. The peak at 287 nm, typical for cell-free extract solution, decreases in all cases, showing a consumption of proteins and/or a change in their structure in the redox reaction. In parallel, a broad band centered at 440 nm appears due to the formation of AgNPs with different sizes. After isolation and re-dispersion of the AgNPs in distilled water, the UV–Vis spectra revealed no wavelength shift but a slight difference in absorption intensities between the samples obtained at different rotation speeds (Figure 8a). A relatively small effect of stirring on the AgNP formation was also observed by EL-Moslamy and coauthors in 24 h experiments using extracellular filtrate of *T*. *harzianum* [49].

More intensive peaks were recorded for the reaction mixtures kept for 144 h (Figure 8b), demonstrating the positive effect of stirring on the rate of AgNP formation. Taking into account the collision theory for the rate of reaction, we propose that an agitation reflects on the orientation of molecules and increases the frequency of effective collisions, thus increasing the rate of reaction. In all cases, the absorption band was observed around 415 nm, indicating that the particle size is not drastically affected by the rotation speed. The silver concentration in aqueous dispersions of AgNPs determined by ICP (Table 2) varies from 11.1 to 15.2 mg/L and from 27.7 to 42.0 mg/L for samples isolated at the 72nd and 144th h, respectively. The amount of AgNPs produced increases with an increase in the rotation speed of agitation until 150 rpm (Table 2). As such, that is why we chose a rotation speed of 150 rpm as representative for the so-called dynamic conditions in our experiments.

**Figure 9:**
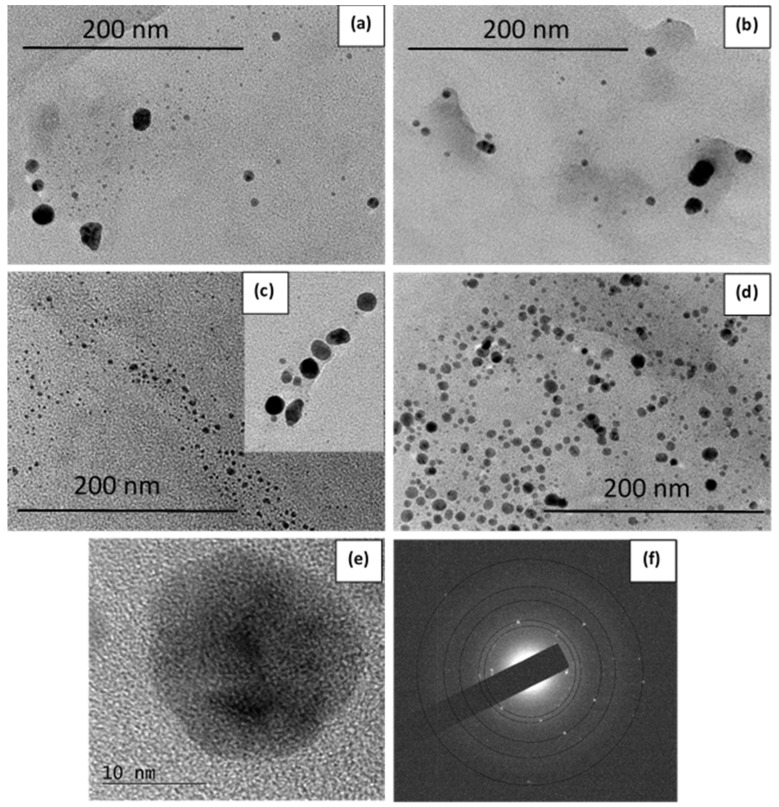
TEM images of the samples (**a**) Ag-72-150 rpm, (**b**) Ag-72-0 rpm, (**c**) Ag-144-150 rpm and (**d**) Ag-144-0 rpm. HR-TEM image of AgNP from the sample Ag-72-150 rpm (**e**) and SAED pattern (**f**).

**Figure 10:**
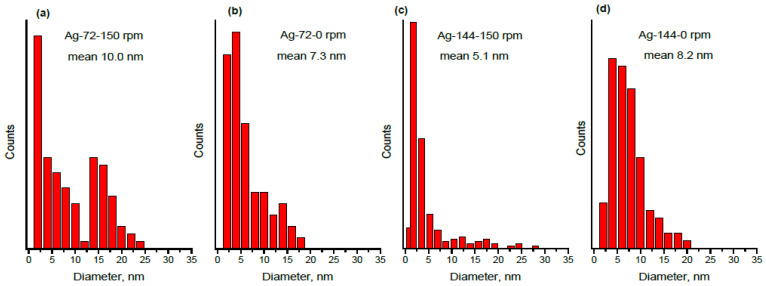
Size distribution for the samples (**a**) Ag-72-150 rpm, (**b**) Ag-72-0 rpm, (**c**) Ag-144-150 rpm and (**d**) Ag-144-0 rpm.

Furthermore, TEM images of four samples, namely, Ag-72-0 rpm and Ag-72-150 rpm, Ag-144-0 rpm and Ag-144-150 rpm, prepared under static and dynamic conditions, for 72 h and 144 h reaction times, respectively, were taken, and these are displayed in Figure 9a–d. The size distribution diagrams of the same samples are shown in Figure 10. The HR-TEM image and the SAED pattern (Figure 9e,f) revealed crystalline AgNPs biosynthesized for 72 h at a rotation speed of 150 rpm. Thus, the influence of stirring on shape, size and size distribution was determined by microscopic analysis. For this purpose, we compare the particle size after 72 h, shown in Figure 9a,b and Figure 10a,b. Under static conditions, there are numerous AgNPs with a size of 2–6 nm, which is most probably due to favorable nucleation and a slower growth of AgNPs affected by the surrounding biomolecules. At agitation of the reaction mixture, the growth process is facilitated, and a significant fraction of particles with a size of 14–16 nm can be observed. When the suspension is kept for a longer time (144 h) without mixing, the particle size under static conditions gradually increases, and the main part of the counted particles shows a diameter of 4–15 nm. Surprisingly, mixing for 144 h leads to a decrease in the mean size, and the core fraction is in the range of 2–6 nm in diameter. In order to confirm this observation, we performed a DLS analysis of samples Ag-144-0 rpm and Ag-144–150 rpm. The DLS-determined Z-average particle size (hydrodynamic size) of AgNPs is 190.8 ± 6.0 nm and 116.8 ± 1.7 nm for Ag-144-0 rpm and Ag-144-150 rpm, respectively, showing the formation of bigger particles under static conditions (Table 3). Not only could the growth of particles happen with time, but the hydrated layer of metallic particles could also become larger. Due to the solvation effect of the capping molecules around the AgNPs, the Z-average size is much higher than the one determined from the TEM images (8.2 and 5.1 nm for Ag-144-0 rpm and Ag-144-150 rpm, respectively, Figure 10c,d). Additionally, the polydispersity index (PDI) values for samples Ag-144-0 rpm and Ag-144-150 rpm are 0.36 ± 0.01 and 0.43 ± 0.02, respectively (Table 3), indicating that the particles can be considered as monodispersed and not aggregated, which is in good agreement with the size distribution derived from the TEM data [62]. Some authors reported that during AgNP synthesis by reduction with NaBH_4_ and the presence of nanocellulose, the mixing of reagents causes various sizes and shapes of AgNPs, while without mixing, only spherical NPs are formed [48]. In our case, we did not observe different particle shapes obtained with and without agitation, and only spherical ones can be seen in the TEM images (Figure 9).

By using the DLS method, the zeta potential was also determined (Table 3). The magnitude of the zeta potential allows one to assess the stability of AgNPs in aqueous colloids. If the zeta potential is more negative than −30 mV (or more positive than +30 mV), the particles are considered stable, as they will repel each other [63]. The zeta potential of samples Ag-144-0 rpm and Ag-144-150 rpm was found to be −49.0 ± 3.0 mV and −51.4 ± 4.3 mV, respectively. Thus, these values are indicative of the high stability of the obtained nanoparticle colloids in both cases (with or without stirring). The negative sign of the zeta potential is due to the presence of negatively charged functional groups in the biomolecules surrounding the AgNPs. Similar negative values of zeta potential were reported for AgNPs coated with organic molecules and prepared by other green methods [48,60,62].

**Figure 11:**
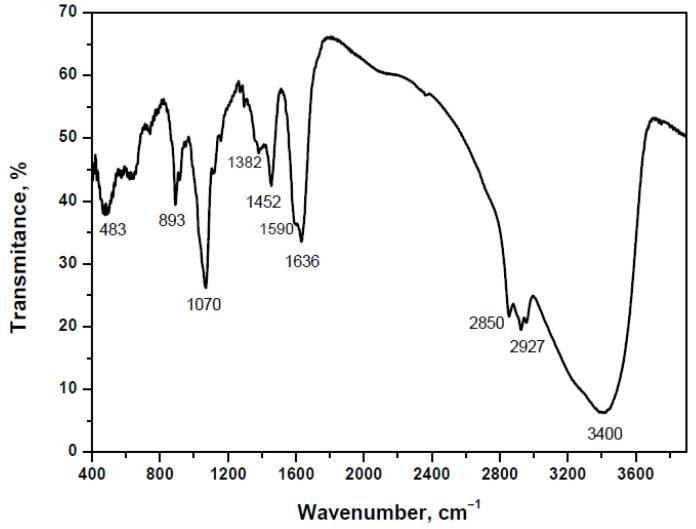
FTIR spectrum of the isolated and dried AgNPs.

In the FTIR spectrum of the AgNPs, shown in Figure 11, stretching vibrations for the deprotonated carboxyl group (ν_COO_^−^) are observed at 1590 and 1382 cm^−1^. Furthermore, the broad band centered at 3400 cm^−1^ is assigned to the stretching vibrations of –N–H and –O–H bonds; the strong bands at 1636 and 1070 cm^−1^ are due to a carbonyl group (ν_C=O_) in amide and a C–O single bond (ν_C–O_); and the weak bands at 2927, 2850 and 1452 cm^−1^ arise from asymmetric and symmetric stretch vibrations and the deformation vibrations of C–H bonds from CH_3_ and CH_2_ groups [64]. Thus, it was suggested that the deprotonated proteins wrap the AgNPs to improve their stability and dispersion in aqueous solutions [49,51]. In our previous work, the –COOH, –NH_2_ and –OH functional groups in the proteins of cell-free extract were also proven by FTIR [56].

### 3.3. Summary of the Optimized Experimental Conditions for AgNP Biosynthesis by Cell-Free Extract from Trichoderma reesei Fungus

It is well known that time, temperature, the concentration of the reactants etc., are important factors for variations in the size and shape of nanoparticles, determining the properties, such as optical, catalytic and antibacterial properties, of materials. As such, this motivated us to explore and optimize as many reaction conditions as possible to achieve control of AgNP formation. The effect of the nutrient medium components for *T*. *reesei* cultivation on the biosynthesis of AgNPs from Ag^+^ ions was previously tested in five media. The basic medium (1) included 2% glucose, 0.1% NH_4_Cl, 0.03% CO(NH_2_)_2_, 0.14% (NH_4_)_2_SO_4_, 0.2% KH_2_PO_4_, 0.03% MgSO_4_·7H_2_O and 0.04% CaCl_2_·2H_2_O. In the other media, four different additives were used, and the contents are as follows: (2) medium 1 + 0.1% yeast extract, (3) medium 1 + 0.1% corn steep liquor, (4) medium 1 + 0.1% peptone medium, and (5) medium 1 + 0.1% casamino acids [55]. It was found that medium 3, containing corn steep liquor, was the most suitable for the cultivation of *T. reesei*, and it was used further for AgNP biosynthesis.

Among the tested parameters, the amount of biomass (5, 10 and 15%) used to obtain the CFE, the initial concentration of the AgNO_3_ solution (2, 10 and 18 mM), the time (up to the 168th h), the optimal conditions of 10% biomass in CFE extraction, 10 mM AgNO_3_ solution and the reaction time of 144 h were chosen for Ag^+^ reduction and the production of AgNPs [56].

In the present paper, we show the results of the effect of stirring rate and temperature on AgNP biosynthesis. We assume a rotation speed of 150 rpm, temperature of 40 °C and biosynthesis duration of 144 h as optimal conditions for producing the maximum amount of NPs.

### 3.4. Antibacterial Activity of Synthesized AgNPs

Some authors presented data on the antibacterial effect of silver nanoparticles on test microorganisms, including biotransformation performed with *Trichoderma* species [43], *T. atroviride* [44], *T. harzianum* [45] and *T. koningii* [46]. Our study focuses on the influence of the experimental conditions on the physicochemical characteristics of the synthesized AgNPs and, consequently, on their inhibitory effect on the growth of *E. coli*, strain 3398, as a test microorganism. Antibacterial tests were conducted with samples Ag-144-0 rpm and Ag-144-150 rpm synthesized at the optimized conditions mentioned above under static and stirring conditions.

**Figure 12:**
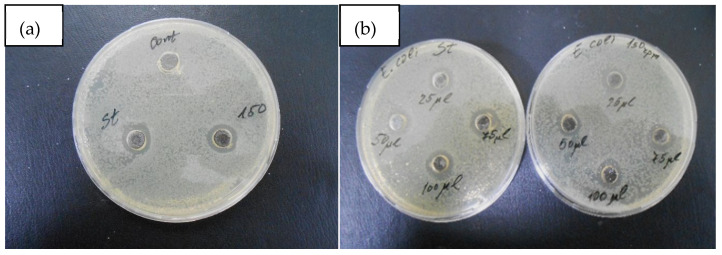
Inhibition zones of the AgNPs obtained by biotransformation under static and dynamic conditions, with respect to *E. coli* strain 3398: (**a**) 50 μL suspension of Ag-144-0 rpm (on the left) and Ag-144-150 rpm (on the right) with distilled water used as control; (**b**) 25, 50, 75 and 100 μL suspension of Ag-144-0 rpm (in the left Petri dish) and Ag-144-150 rpm (in the right Petri dish).

The results on the antibacterial effect of the synthesized AgNPs are presented in Table 4 and Figure 12.

As can be seen in Table 4, increasing the applied amount of AgNPs caused an increase in the antibacterial effect against the tested microorganism. At the maximum amount used (100 μL), the size of the inhibition zones was 14.5 mm for both cases. The data presented show that the antibacterial effect of AgNPs is reproducible, and it is not affected by the size and morphology of the AgNPs obtained under different conditions of mass transfer.

## 4. Conclusions

In conclusion, it can be stated that the CFE of *T. reesei* is a suitable reagent for the production of monodispersed AgNPs, which are stabilized by the capping effect of the biomolecules in CFE. The study of temperature dependence revealed that, under optimized conditions for biotransformation, the highest amount of stable sphere-like AgNPs was produced at 40 °C.

When the mass transfer effect was studied under static and dynamic mixing conditions, a rotation speed of 150 rpm was chosen as the most suitable for the biosynthesis of stable and small AgNPs in the highest amount.

The TEM analysis showed that the size of AgNPs was in the range of 2–25 nm, while the main fraction of the NPs had a diameter of 2–6 nm.

The AgNPs capped with biomolecules demonstrated antibacterial activity toward *E. coli.* The tests showed no significant difference between AgNPs synthesized under static conditions and those under stirring conditions with a speed of 150 rpm.

Overall, taking into account our investigations on reaction conditions, we can conclude that the optimal settings for the synthesis of stable spherical monodispersed AgNPs are (a) *T. reesei* cultivation in a glucose-based medium containing corn steep liquor, (b) 10% biomass to extract fungus metabolites and enzymes (CFE), (c) a 10 mM concentration of AgNO_3_, (d) a temperature of 40 °C, (f) a stirring speed of 150 rpm and (g) a reaction time of 144 h.

## Figures and Tables

**Table 1:** Sample notation.

Sample	Description	Sample	Description	Conditions
Ag-72-4 °C	AgNPs synthesized for 72 h at 4 °C	Ag-72-0 rpm	AgNPs synthesized for 72 h at 30 °C without stirring	Static
Ag-72-20 °C	AgNPs synthesized for 72 h at 20 °C	Ag-72-50 rpm	AgNPs synthesized for 72 h at 30 °C at stirring 50 rpm	Dynamic
Ag-72-30 °C	AgNPs synthesized for 72 h at 30 °C	Ag-72-150 rpm	AgNPs synthesized for 72 h at 30 °C at stirring 150 rpm	Dynamic
Ag-72-40 °C	AgNPs synthesized for 72 h at 40 °C	Ag-72-250 rpm	AgNPs synthesized for 72 h at 30 °C at stirring 250 rpm	Dynamic
Ag-144-4 °C	AgNPs synthesized for 144 h at 4 °C	Ag-144-0 rpm	AgNPs synthesized for 144 h at 30 °C without stirring	Static
Ag-144-20 °C	AgNPs synthesized for 144 h at 20 °C	Ag-144-50 rpm	AgNPs synthesized for 144 h at 30 °C at stirring 50 rpm	Dynamic
Ag-144-30 °C	AgNPs synthesized for 144 h at 30 °C	Ag-144-150 rpm	AgNPs synthesized for 144 h at 30 °C at stirring 150 rpm	Dynamic
Ag-144-40 °C	AgNPs synthesized for 144 h at 40 °C	Ag-144-250 rpm	AgNPs synthesized for 144 h at 30 °C at stirring 250 rpm	Dynamic

**Table 2:** Silver concentration of aqueous dispersions of the AgNPs after their isolation determined by ICP.

Sample	Ag (mg/L)	Sample	Ag (mg/L)
Ag-72-20 °C	13.6	Ag-144-20 °C	31.9
Ag-72-30 °C	14.5	Ag-144-30 °C	32.5
Ag-72-40 °C	22.0	Ag-144-40 °C	61.1
Ag-72-0 rpm	11.1	Ag-144-0 rpm	31.9
Ag-72-50 rpm	10.8	Ag-144-50 rpm	27.7
Ag-72-150 rpm	15.2	Ag-144-150 rpm	42.0
Ag-72-250 rpm	14.2	Ag-144-250 rpm	35.6

**Table 3:** DLS data for the AgNPs obtained under static and dynamic conditions.

Sample	Zeta Potential (mV)	Z-Average Particle Size (nm)	PDI
Ag-144-0 rpm	−49.0 ± 3.0	190.8 ± 6.0	0.36 ± 0.01
Ag-144-150 rpm	−51.4 ± 4.3	116.8 ± 1.7	0.43 ± 0.02

**Table 4:** Inhibition zones of the AgNPs (25, 50, 75 and 100 μL suspensions) obtained by biotransformation under static (Ag-144-0 rpm) and dynamic (Ag-144-150 rpm) conditions, with respect to *E. coli* strain 3398.

Volume of AgNPs Suspension (μL)	Inhibition Zone of Ag-144-0 rpm (mm)	Inhibition Zone of Ag-144-150 rpm (mm)
25	11.5	11.0
50	12.5	12.0
75	13.5	13.0
100	14.5	14.5

## Data Availability

The data presented in this study are available upon request by contact with the corresponding author.

## References

[1] Rai M., Birla S., Ingle A.P., Gupta I., Gade A., Abd-Elsalam K., Marcato P.D., Duran N. (2014). Nanosilver: An inorganic nanoparticle with myriad potential applications. Nanotechnol. Rev..

[2] Haider A., Kang I.K. (2015). Preparation of silver nanoparticles and their industrial and biomedical applications: A comprehensive review. Adv. Mater. Sci. Eng..

[3] Singh J., Kaur G., Kaur P., Bajaj R., Rawat M. (2016). A review on green synthesis and characterization of silver nanoparticles and their applications: A green nanoworld. World J. Pharm. Pharmaceut. Sci..

[4] Verma P., Maheshwari S.K. (2019). Applications of silver nanoparticles in diverse sectors. Int. J. Nano Dimens..

[5] Mariselvam R., Ranjitsingh A.J.A., Nanthini A.U.R., Kalirajan K., Padmalatha C., Selvakumar P.M. (2014). Green synthesis of silver nanoparticles from the extract of the inflorescence of *Cocos nucifera* (Family: Arecaceae) for enhanced antibacterial activity. Spectrochim. Acta A Mol. Biomol. Spectrosc..

[6] Jeeva K., Thiyagarajan M., Elangovan V., Geetha N., Venkatachalam P. (2014). *Caesalpinia coriaria* leaf extracts mediated biosynthesis of metallic silver nanoparticles and their antibacterial activity against clinically isolated pathogens. Ind. Crops Prod..

[7] Panácek A., Kolár M., Vecerová R., Prucek R., Soukupová J., Krystof V., Hamal P., Zboril R., Kvítek L. (2009). Antifungal activity of silver nanoparticles against *Candida* spp. Antifungal activity of silver nanoparticles against *Candida* spp.. Biomaterials.

[8] Galdiero S., Falanga A., Vitiello M., Cantisani M., Marra V., Galdiero M. (2011). Silver Nanoparticles as Potential Antiviral Agents. Molecules.

[9] Sriramulu M., Sumathi S. (2017). Photocatalytic, antioxidant, antibacterial and antiinflammatory activity of silver nanoparticles synthesised using forest and edible mushroom. Adv. Nat. Sci. Nanosci. Nanotechnol..

[10] Saeed B.A., Lim V., Yusof N.A., Khor K.Z., Rahman H.S., Samad N.A. (2019). Antiangiogenic properties of nanoparticles: A systematic review. Int. J. Nanomed..

[11] Buttacavoli M., Albanese N.N., Cara G.D., Alduina R., Faleri C., Gallo M., Pizzolanti G., Gallo G., Feo S., Baldi F. (2018). Anticancer activity of biogenerated silver nanoparticles: An integrated proteomic investigation. Oncotarget.

[12] Mehnath S., Das A.K., Vermac S.K., Jeyaraj M., Verma S.K., Das A.K. (2021). Biosynthesized/greensynthesized nanomaterials as potential vehicles for delivery of antibiotics/drugs. Biosynthesized Nanomaterials—Comprehensive Analytical Chemistry.

[13] Uttayarat P., Eamsiri J., Tangthong T., Suwanmala P. (2015). Radiolytic synthesis of colloidal silver nanoparticles for antibacterial wound dressings. Adv. Mater. Sci. Eng..

[14] Khayati G.R., Janghorban K. (2012). The nanostructure evolution of Ag powder synthesized by high energy ball milling. Adv. Powder Technol..

[15] Harra J., Mäkitalo J., Siikanen R., Virkki M., Genty G., Kobayashi T., Kauranen M., Mäkelä J.M. (2012). Size-controlled aerosol synthesis of silver nanoparticles for plasmonic materials. J. Nanopart. Res..

[16] Yin H., Yamamoto T., Wada Y., Yanagida S. (2004). Large-scale and size-controlled synthesis of silver nanoparticles under microwave irradiation. Mater. Chem. Phys..

[17] Rheima A.M., Mohammed M.A., Jaber S.H., Shahad Abbas Hameed S.A. (2019). Synthesis of silver nanoparticles using the UV-irradiation technique in an antibacterial application. J. Southwest Jiaotong Univ..

[18] Iravani S., Korbekandi H., Mirmohammadi S.V., Zolfaghari B. (2014). Synthesis of silver nanoparticles: Chemical, physical and biological methods. Res. Pharm. Sci..

[19] Tien D.C., Tseng K.H., Liao C.Y., Huang J.C., Tsung T.T. (2008). Discovery of ionic silver in silver nanoparticle suspension fabricated by arc discharge method. J. Alloys Compd..

[20] Borra J.-P., Jidenko N., Hou J., Weber A. (2015). Vaporization of bulk metals into single-digit nanoparticles by non-thermal plasma filaments in atmospheric pressure dielectric barrier discharges. J. Aerosol Sci..

[21] Tsuji T., Iryo K., Watanabe N., Tsuji M. (2002). Preparation of silver nanoparticles by laser ablation in solution: Influence of laser wavelength on particle size. Appl. Surf. Sci..

[22] Guzmán M.G., Dille J., Godet S. (2009). Synthesis of silver nanoparticles by chemical reduction method and their antibacterial activity. Int. J. Chem. Biomol. Eng..

[23] Quintero-Quiroz C., Acevedo N., Zapata-Giraldo J., Botero L.E., Quintero J., Zárate-Triviño D., Saldarriaga J., Pérez V.Z. (2019). Optimization of silver nanoparticle synthesis by chemical reduction and evaluation of its antimicrobial and toxic activity. Biomater. Res..

[24] Zhang Y., Chen F., Zhuang J., Tang Y., Wang D., Wang Y., Dong A., Ren N. (2002). Synthesis of silver nanoparticles via electrochemical reduction on compact zeolite film modified electrodes. Chem. Commun..

[25] Fu X., Yu W., Lin Y., Wang D., Shi H., Yan F. (2005). Preparation of Concentrated Stable Fluids Containing Silver Nanoparticles in Nonpolar Organic Solvent. J. Dispers. Sci. Technol..

[26] Hah H.J., Koo S.M., Lee S.H. (2003). Preparation of Silver Nanoparticles through Alcohol Reduction with Organoalkoxysilanes. J. Sol-Gel Sci. Technol..

[27] Zhang W., Qiao X., Chen J. (2007). Synthesis of nanosilver colloidal particles in water/oil microemulsion. Colloids Surf. A Physicochem. Eng. Aspects..

[28] Mănoiu V.S., Aloman A. (2010). Obtaining silver nanoparticles by sonochemical methods. U.P.B. Sci. Bull. Ser. B.

[29] Rauwel P., Küünal S., Ferdov S., Rauwel E. (2015). A review on the green synthesis of silver nanoparticles and their morphologies studied via TEM. Adv. Mater. Sci. Eng..

[30] Ahmed S., Ahmad M., Swami B.L., Ikram S. (2016). A review on plants extract mediated synthesis of silver nanoparticles for antimicrobial applications: A green expertise. J. Adv. Res..

[31] Rajeshkumar S., Bharath L.V. (2017). Mechanism of plant-mediated synthesis of silver nanoparticles—A review on biomolecules involved, characterisation and antibacterial activity. Chem.-Biol. Interact..

[32] Das R.K., Pachapur V.L., Lonappan L., Naghdi M., Pulicharla R., Maiti S., Cledon M., Dalila L.M.A., Sarma S.J., Brar S.K. (2017). Biological synthesis of metallic nanoparticles: Plants, animals and microbial aspects. Nanotechnol. Environ. Eng..

[33] Corciova A., Ivanescu B. (2018). Biosynthesis, characterization and therapeutic applications of plant-mediated silver nanoparticles. J. Serb. Chem. Soc..

[34] Javaid A., Oloketuyi S.F., Khan M.M., Khan F. (2018). Diversity of bacterial synthesis of silver nanoparticles. BioNanoScience.

[35] Lahiri D., Nag M., Ray R.R., Ghosh S., Webster T. (2021). Mycosynthesis of silver nanoparticles: Mechanism and applications. Nanobiotechnology—Microbes and Plant Assisted Synthesis of Nanoparticles, Mechanisms and Applications.

[36] Khan A.U., Malik N., Khan M., Cho M.H., Khan M.M. (2018). Fungi-assisted silver nanoparticle synthesis and their applications. Bioprocess Biosyst. Eng..

[37] Khandel P., Shahi S.K. (2018). Mycogenic nanoparticles and their bio-prospective applications: Current status and future challenges. J. Nanostruct. Chem..

[38] Ahmad A., Mukherjee P., Senapati S., Mandal D., Khan M.I., Kumar R., Sastry M. (2003). Extracellular biosynthesis of silver nanoparticles using the fungus *Fusarium oxysporum*. Colloids Surf. B.

[39] Tripathi R.M., Gupta R.K., Shrivastav A., Singh M.P., Shrivastav B.R., Singh P. (2013). *Trichoderma koningii* assisted biogenic synthesis of silver nanoparticles and evaluation of their antibacterial activity. Adv. Nat. Sci. Nanosci. Nanotechnol..

[40] Kashyap P.L., Kumar S., Srivastava A.K., Sharma A.K. (2013). Myco nanotechnology in agriculture: A perspective. World J. Microb. Biot..

[41] Vahabi K., Dorcheh S.K., Gupta V.K., Schmoll M., Herrera-Estrella A., Upadhyay R.S., Druzhinina I., Tuohy M.G. (2014). Biosynthesis of silver nano-particles by *Trichoderma* and its medical applications. Biotechnology and Biology of Trichoderma.

[42] Mukherjee P., Roy M., Mandal B.P., Dey G.K., Mukherjee P.K., Ghatak J., Tyagi A.K., Kale S.P. (2008). Green synthesis of highly stabilized nanocrystalline silver particles by a non-pathogenic and agriculturally important fungus *T*. *asperellum*. Nanotechnology.

[43] Elgorban A.M., Al-Rahmah A.N., Sayed S.R., Hirad A., Mostafa A.A.F., Bahkali A.H. (2016). Antimicrobial activity and green synthesis of silver nanoparticles using *Trichoderma viride*. Biotechnol. Biotec. Equip..

[44] Saravanakumar K., Wang M.H. (2018). *Trichoderma* based synthesis of anti-pathogenic silver nanoparticles and their characterization, antioxidant and cytotoxicity properties. Microb. Pathog..

[45] Ahluwalia V., Kumar J., Sisodia R., Shakil N.A., Walia S. (2014). Green synthesis of silver nanoparticles by *Trichoderma harzianum* and their bio-efficacy evaluation against *Staphylococcus aureus* and *Klebsiella pneumonia*. Ind. Crop. Prod..

[46] El-Wakil D.A. (2020). Antifungal Activity of Silver Nanoparticles by Trichoderma species: Synthesis, Characterization and Biological Evaluation. Egypt. J. Phytopathol..

[47] Oksanen T., Pere J., Paavilainen L., Buchert J., Viikari L. (2000). Treatment of recycled kraft pulps with *Trichoderma reesei* kemicellulases and cellulases. J. Biotechnol..

[48] Cieśla J., Chylińska M., Zdunek A., Szymańska-Chargot M. (2020). Effect of different conditions of synthesis on properties of silver nanoparticles stabilized by nanocellulose from carrot pomace. Carbohyd. Polym..

[49] EL-Moslamy S.H., Elkady M.F., Rezk A.H., Abdel-Fattah Y.R. (2017). Applying Taguchi design and largescale strategy for mycosynthesis of nano-silver from endophytic Trichoderma harzianum SYA.F4 and its application against phytopathogens. Sci. Rep..

[50] Balakumaran M.D., Ramachandran R., Balashanmugam P., Mukeshkumar D.J., Kalaichelvan P.T. (2016). Mycosynthesis of silver and gold nanoparticles: Optimization, characterization and antimicrobial activity against human pathogens. Microbiol. Res..

[51] Ma L., Su W., Liu J.X., Zeng X.X., Huang Z., Li W., Liu Z.C., Tang J.X. (2017). Optimization for extracellular biosynthesis of silver nanoparticles by *Penicillium aculeatum* Su1 and their antimicrobial activity and cytotoxic effect compared with silver ions. Mater. Sci. Eng. C Mater. Biol. Appl..

[52] Fayaz A.M., Balaji K., Kalaichelvan P.T., Venkatesan R. (2009). Fungal based synthesis of silver nanoparticles—An effect of temperature on the size of particles. Colloids Surf. B Biointerfaces.

[53] Saxena J., Sharma P.K., Sharma M.M., Singh A. (2016). Process optimization for green synthesis of silver nanoparticles by *Sclerotinia sclerotiorum* MTCC 8785 and evaluation of its antibacterial Properties. SpringerPlus.

[54] Rahim K.A., Mahmoud S.Y., Ali A.M., Almaary K.S., Mustafa A.E.Z., Husseiny S.M. (2017). Extracellular biosynthesis of silver nanoparticles using *Rhizopus stolonifer*. Saudi J. Biol. Sci..

[55] Gemishev O.T., Panayotova M.I., Panayotov V.T. (2021). Biosynthesis of silver nanoparticles by cell-free extract from *Trichoderma reesei*—Study on the influence of growth media. IOP Conf. Ser.-Mat. Sci. Eng..

[56] Gemishev O.T., Panayotova M.I., Mintcheva N.N., Djerahov L.P., Tyuliev G.T., Gicheva G.D. (2019). A green approach for silver nanoparticles preparation by cell-free extract from *Trichoderma reesei* fungi and their characterization. Mater. Res. Express.

[57] Janas P., Targoński Z. (1995). Effect of temperature on the production of cellulases, xylanases and lytic enzymes by selected *Trichoderma reesei* mutants. Acta Microbiol..

[58] Chokhawala H.A., Roche C.M., Kim T.W., Atreya M.E., Vegesna N., Dana C.M., Blanch H.W., Clark D.S. (2015). Mutagenesis of *Trichoderma reesei* endoglucanase I: Impact of expression host on activity and stability at elevated temperatures. BMC Biotechnol..

[59] Lee S.H., Jun B.H. (2019). Silver Nanoparticles: Sythesis and application for nanomedicine. Int. J. Mol. Sci..

[60] Rolim W.R., Pelegrino M.T., Lima B.A., Ferraz L.S., Costa F.N., Bernardes J.S., Rodigues T., Brocchi M., Seabra A.B. (2019). Green tea extract mediated biogenic synthesis of silver nanoparticles: Characterization, cytotoxicity evaluation and antibacterial activity. Appl. Surf. Sci..

[61] Liu H., Zhang H., Wang J., Wei J. (2020). Effect of temperature on the size of biosynthesized silver nanoparticle: Deep insight into microscopic kinetics analysis. Arab. J. Chem..

[62] Danaei M., Dehghankhold M., Ataei S., Davarani F.H., Javanmard R., Dokhani A., Khorasani S., Mozafari M.R. (2018). Impact of particle size and polydispersity index on the clinical applications of lipidic nanocarrier systems. Pharmaceutics.

[63] Fernández J.G., Fernández-Baldoc M.A., Berni E., Camí G., Duránd N., Raba J., Sanz M.I. (2016). Production of silver nanoparticles using yeasts and evaluation of their antifungal activity against phytopathogenic fungi. Process Biochem..

[64] Coates J., Meyers R.A. (2000). Interpretation of Infrared Spectra, a Practical Approach. Encyclopedia of Analytical Chemistry.

